# Dolichocephaly, Arachnodactyly, Diplopia, and Distal Myopathy – Novel Phenotype of MICU1 Variant c.553C>T

**DOI:** 10.7759/cureus.52672

**Published:** 2024-01-21

**Authors:** Josef Finsterer, Awini Barwari

**Affiliations:** 1 Neurology, Neurology and Neurophysiology Center, Vienna, AUT

**Keywords:** ataxia, myopathy, cognitive impairment, micu1, mutation

## Abstract

Pathogenic variants in mitochondrial calcium uptake 1 (*MICU1)* manifest phenotypically heterogeneously but most frequently in the brain and skeletal muscle. Dolichocephaly, arachnodactyly, diplopia, and distal myopathy have not been reported in carriers of a pathogenic *MICU1* variant. The patient is a 23-year-old female with consanguineous parents (first cousins) who was a carrier of the homozygous *MICU1* variant c.553C>T, phenotypically presenting with developmental delay, intellectual disability, ataxia, dysmorphia (dolichocephaly, arachnodactyly, clinodactyly, hypertelorism, wide nasal bridge), myopathy (ptosis, double vision, strabismus, distal limb weakness, diffuse wasting, hypotonia), hyperextensible joints and hyperkyphosis. Features not previously described were dolichocephaly, arachnodactyly, broad nasal bridge, double vision, and distal myopathy. She was treated with physical therapy, speech therapy, and occupational therapy and received escitalopram and mirtazapine for concomitant depression, anxiety disorder, and insomnia. The presented case shows that the phenotypic heterogeneity of pathogenic *MICU1 *variants is even greater than previously assumed. Treatment of *MICU1*-related phenotypes is symptomatic, but these patients benefit from physical therapy, behavioral therapy, speech therapy, and antidepressant treatment.

## Introduction

The mitochondrial calcium uptake-1 (MICU1) gene (OMIM #605084), also known as the CALC, EFHA3, MPXPS, or CBARA1 gene, is located on chromosome 10q22.1 and includes 17 exons [1,2,3]. MICU1 encodes the gatekeeper of the mitochondrial calcium uniporter protein, which is located in the inner mitochondrial membrane [4]. The MICU1 protein functions as a calcium uniporter protein and is responsible for delivering calcium ions to the mitochondrial matrix [5]. Pathogenic variants in MICU1 manifest phenotypically with multisystem disease affecting the skeletal muscles (proximal myopathy, ptosis, ophthalmoparesis, dysarthria, scapula alata), the brain (cognitive impairment, mental retardation, attention deficit, depression, insomnia, seizures, extrapyramidal abnormalities (chorea, dystonia, athetosis, tremor, orofacial dyskinesia, tics), ataxia, nystagmus, dysarthria, polymicrogyria, dysmorphic basal ganglia, hypoplastic anterior limbs of internal capsules, cerebellar dysplasia, optic atrophy), the peripheral nerves (axonal neuropathy), the heart (dilated cardiomyopathy), the skeleton (microcephaly, hypertelorism, high arched palate, pes cavus, clinodactyly, hyperlordosis, hyperkyphosis, radioulnar synostosis, low-set ears), the endocrine organs (short stature, hypothyroidism), and the eyes (cataract) [5-16]. The most common phenotype is an autosomal recessive, childhood-onset myopathy with proximal muscle weakness with extrapyramidal signs (MPXPS) (OMIM #615673) [15]. Here, we report a patient with a MICU1 variant that manifested phenotypically with a number of novel features.

## Case presentation

The patient is a 23-year-old female, height 165 cm, weight 36 kg, who was diagnosed with mitochondrial myopathy due to the homozygous variant c.553C>T in exon 6 of the MICU1 gene. The patient presented with developmental delay at the age of three and cognitive impairment that manifested first in elementary school (Table 1). For this reason, she moved to a special school, which she attended for the next 12 years with below-average academic performance. At the age of 12, she developed gait disturbance due to weakness in the right lower extremities. Her medical history was also positive for recurrent abdominal pain, but clinical examination, blood tests, and abdominal ultrasound were repeatedly normal. Family history revealed no evidence of cerebral, muscle, or cardiac disease, but her parents were first-degree cousins. The parents, brother, and two sisters were phenotypically unaffected.

**Table 1:** Patients carrying pathogenic MICU1 variants reported until the end of 2023 CI: cognitive impairment, dCMP: dilated cardiomyopathy, EPS: extra-pyramidal syndrome, HYP: hypotonia, MP: myopathy, PSH: pseudohypertrophy, SS: short stature

Age	Sex	Phenotype	Genotype	Reference
23	f	MP, CI, dolichocephaly, arachnodactyly, strabismus	c.553C>T	[index case]
5	f	motor/speech delay, ataxia, insomnia, attention ¯, MP, clinodactyly, HYP, hyperlordosis, CI	c.553C>T	[6]
5	m	MP, restlessness, ataxia, tip-toe walking, sensitivity ¯	c.52.C>T, Ex 2del	[6]
17	m	proximal MP, PSH, CI, high arched palate, dystonia, chorea, pes cavus, dysarthria	c.1072-1G>C	[5]
44	m	CI, ataxia, EPS, strabismus, MP	c.385C>T	[7]
3.5	m	severe episodic muscle weakness, MP, dyslalia, dysarthria, PSH	Ex 2del	[8]
2-23	8m/5f	SS, CI, ataxia, EPS, motor delay, MP, microcephaly, PSH	p-Q185X, Ex9-10del	[9]
5	f	MP, EPS, dCMP, speech delay, PSH	c.1295delA	[10]
2	f	MP, speech delay	c.1295delA	[10]
1.5-5	n=11	SS, proximal MP, CI, EPS	c.1078-1G>C	[11]
1-8	n=4	SS, MP	c.741-1G>A	[11]
3	f	polymicrogyria, dysmorphic basal ganglia, cerebellar dysplasia, white matter lesions	c.161+1G>A, c.386G>C	[12]
13	f	MP, EPS	5UTR.Ex1del	[13]
12	m	nystagmus, motor delay, learning disability, MP, HYP, cataract	5UTR.Ex1del	[13]
3	f	motor + speech delay, learning disability, MP, ataxia, clinodactyly	p.Q185X	[14]
52	m	segmental dystonia, seizures, CI, hypothyroidism, cleft palate short stature, facial dysmorphism, radioulnar synostosis, dysarthria	c.1078-1G>C	[15]
53	m	developmental delay, MP, axonal neuropathy	c.1078-1G>C	[15]
7	f	MP, ataxia, learning disability	c.741-1G>A	[16]
10	f	MP, ataxia, learning disability	c.741-1G>A	[16]

Clinical neurological examination at the age of 14 years revealed startleness but no hyperekplexia, social withdrawal, avoidance of eye contact, general wasting, and hyperkyphosis of the thoracic spine. Blood tests revealed recurrent mild elevation of creatine-kinase. Needle electromyography was normal in muscles with and without weakness. Nerve conduction studies were normal. A muscle biopsy from the left biceps muscle revealed only nonspecific changes. The cerebral CT was normal. The EEG only showed increased background slowing (diffuse, intermittent theta waves without drowsiness). Neuropsychological testing revealed adjustment disorders, anxiety, depression, moderate intellectual disability, a negative self-image, and feelings of being unloved. Genetic testing revealed the homozygous variant c.553C>T in exon 6 of the MICU1 gene.

**Figure 1:**
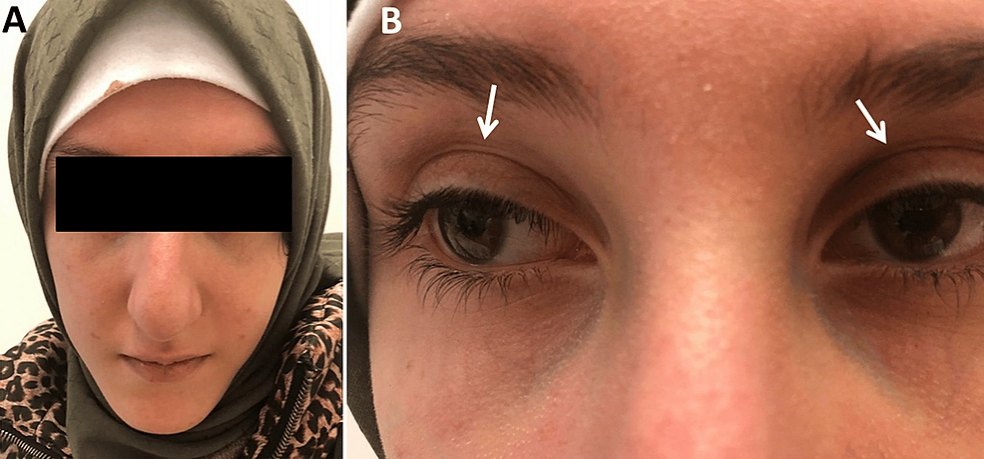
the index patient presented with dsymorphic features such as dolichocephaly (panel A), broad nasal bridge, strabismus, hypertelorism, and mild ptosis (panel B)

**Figure 2:**
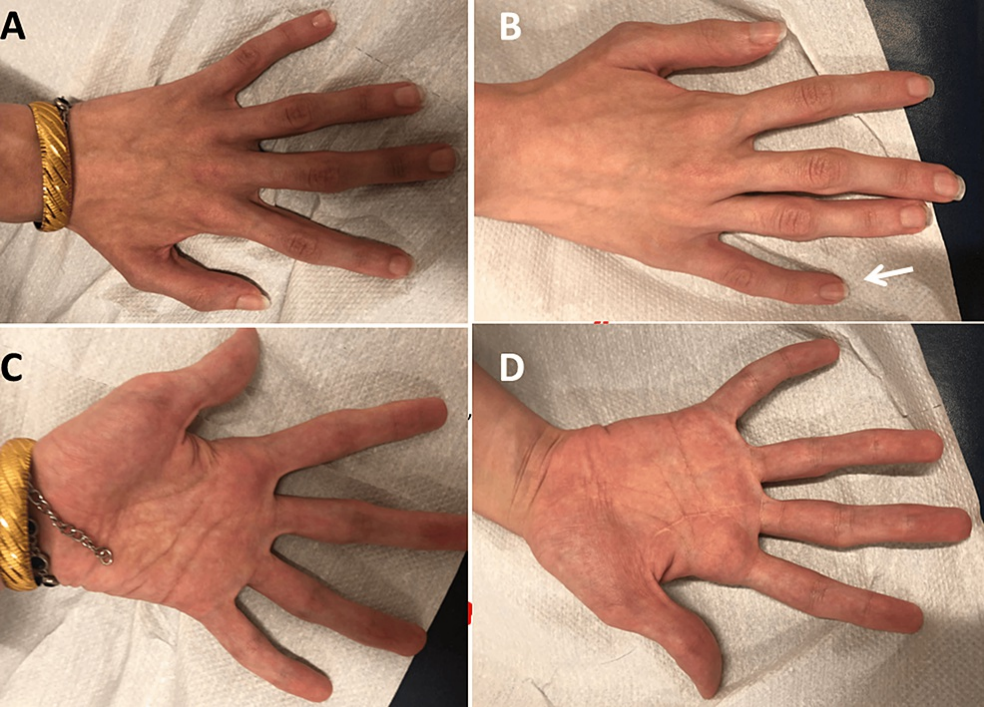
the index patients hands (left hand: panels A and C, right hand: panels B and D) were remarkable for arachnodactyly, clinodactyly, and hyperextensible joints

A second neurological examination was performed at the age of 23 years because the patient reported recurrent diplopia when looking in all directions but without diurnal fluctuations for three years. She presented as a depressed, anxious, taciturn woman who followed instructions correctly but inconsistently. Her face was mildly dysmorphic with dolichocephalus, hypertelorism, strabismus, and mild ptosis bilaterally (Figure 1). She was near-sighted and had significant muscle soreness in her neck. There was no ophthalmoparesis. In the upper limbs, there was distal muscle weakness of the hand and finger muscle (M5-), diffuse wasting, arachnodactyly, clinodactyly (Figure 2), hypotonia, hyperextensible finger and wrist joints, decreased tendon reflexes, and a positive snap reflex on the left side. In the lower limbs, there was discrete dorsiflexion weakness bilaterally, reduced tendon reflexes, and diffuse muscle wasting, but without fasciculations, pseudohypertrophy, or sensory disturbances. She had mild ataxia and had difficulty performing the Unterberger treadmill test correctly and staying on line while walking with her eyes open. It was impossible to walk on the line with her eyes closed. She did not have cachexia. Blood tests revealed erythrocytosis, neutrophilia, lymphopenia, hyperbilirubinemia, mildly elevated transaminases, and mild elevation of creatine-kinase. Tests for vasculitis were normal. Needle electromyography, nerve conduction studies, and repetitive nerve stimulation were normal. Antibodies against acetylcholine receptors and MUSK were negative. MRI of the orbita was normal. Abdominal ultrasound was normal.

Her current medication included escitalopram (10 mg/d), mirtazapine (15 mg/d), and tizanidine (2 mg/d). She benefited from regular physical therapy, speech therapy, and behavioral therapy.

## Discussion

The patient presented is of interest for a mitochondrial multisystem disorder due to the previously reported [6]mutation c.533C>T in MICU1. The patient had some phenotypic features not previously reported in MICU1 mutation carriers (Table 1). Therefore, the case expands the scope of the phenotypic spectrum and highlights that MICU1 variants manifest with broader phenotypic heterogeneity than previously thought. Previously unreported phenotypic features of the index patient included dolichocephaly, arachnodactyly, broad nasal bridge, distal myopathy, and diplopia [5-16]. Even a larger series of MICU1 patients did not report them [9]. Myopathy was diagnosed based on clinical examination (weakness, wasting, decreased tendon reflexes. ptosis), recurrent CK elevations, normal nerve conductions studies, and previous reports showing that myopathy is a common phenotypic feature of MICU1 variants [5-11,13-15]. Normal electromyography and nonspecific muscle biopsy do not rule out myopathy. Diplopia was interpreted as decompensated strabismus as alternative causes were ruled out. These include ophthalmoparesis due to ocular myopathy, myasthenia, myasthenic syndrome, ocular myositis, endocrine orbitopathy, diabetes, vasculitis, retroorbital mass, and cranial nerve neuropathy. Myopathy in MICU1 mutation carriers previously reported began in childhood and, in contrast to the index patient, showed proximally accentuated weakness, closely resembling dystrophinopathies and limb-girdle muscular dystrophies (LGMDs) [5]. Muscle weakness was usually static, while extrapyramidal manifestations progressed. However, reverse trends were also reported during follow‑up [5]. Whether erythrocytosis, neutrophilia, lymphopenia, and hyperbilirubinemia in the index patient were due to the MICU1 variant or due to other causes unrelated to the genetic defect remains speculative. No hematological abnormalities were reported in patients carrying pathogenic MICU1 variants.

Phenotypic features previously reported [5-16] and also present in the index patient included ataxia, developmental delay, cognitive impairment, mental retardation, depression, insomnia, ptosis, strabismus, hypertelorism, clinodactyly, hyperkyphosis, and short stature. The patient had no previously reported features such as seizures, extrapyramidal features, nystagmus, structural abnormalities of the brain on cerebral imaging, proximal myopathy, dysarthria, pseudohypertrophy of the calves, sensory axonal neuropathy, dilated cardiomyopathy, microcephaly, high-arched palate, pes cavus, radioulnar synostosis, low-set ears, hypothyroidism or cataracts [5-16]. The phenotypic heterogeneity in patients carrying pathogenic MICU1 variants is most likely due to the different specific mutations, but it cannot be excluded that additional genetic factors, such as polymorphisms or variants of unknown significance, contribute to the specific clinical presentation. In any case, the multisystem involvement highlights the underlying mitochondrial pathology [9,11,12].

The causative mutation in the index patient has only been described once [6]. The previously reported patient carrying the c.553C>T variant was a 5-year-old female born as the first child of consanguineous Turkish parents. Initially, she suffered from delayed motor milestones, delayed speech development, moderate ataxia with frequent falls, motor restlessness, exercise intolerance, decreased attention, and insomnia with frequent awakenings. At 5 years of age, cognitive impairment, muscle weakness, hypotonia, hyperlordosis, and clinodactyly also occurred [6]. The cerebral MRI was normal. Compared to the index patient, this patient showed similar features.

## Conclusions

The presented case shows that the phenotypic heterogeneity of pathogenic MICU1 variants is even greater than previously assumed. In addition to previously reported phenotypic features, carriers of the c.553C>T variant may exhibit dolichocephaly, arachnodactyly, broad nasal bridge, distal myopathy, and diplopia. Treatment of MICU1-related phenotypes is symptomatic, but these patients benefit from physical therapy, occupational therapy, speech therapy, and antidepressant treatment.
